# Heterogeneity and risk assessment in minor ischemic stroke

**DOI:** 10.3389/fneur.2026.1993509

**Published:** 2026-10-22

**Authors:** Baozhou Xing, Li Zhang, Wen-Jun Tu

**Affiliations:** 1 Clinical Medical College of Shandong Second Medical University, Weifang, China; 2 Department of Neurology, Aerospace Center Hospital, Peking University Aerospace School of Clinical Medicine, Beijing, China

**Keywords:** dual antiplatelet therapy, early neurological deterioration, etiologic subtype, large-vessel occlusion, minor ischemic stroke, risk stratification, thrombolysis

## Abstract

Minor ischemic stroke is often defined by a low National Institutes of Health Stroke Scale (NIHSS) score, yet patients with similar scores may have very different vascular lesions, tissue injury, and stroke mechanisms. This narrative review examines four domains that modify prognosis and management: symptom relevance, vascular and hemodynamic status, tissue status, and etiologic subtype. Adverse trajectories differ in timing. Early neurological deterioration (END) or infarct growth usually occurs within hours to days, recurrent ischemia clusters over days to weeks, and incomplete recovery may persist for months. A low NIHSS score may occur despite a disabling posterior-circulation or eloquent deficit, large- or medium-vessel occlusion, poor collateral flow, hypoperfusion, branch atheromatous disease, or an active embolic source. These outcomes are distinct from the mechanisms that drive them; high-risk minor stroke is not limited to large-vessel occlusion. PRISMS did not demonstrate benefit of alteplase over aspirin in selected patients with minor nondisabling stroke, ARAMIS found dual antiplatelet therapy noninferior to alteplase in a similarly selected population, and TEMPO-2 did not support routine tenecteplase in minor stroke selected by intracranial occlusion or focal perfusion abnormality. At ESOC 2026, MILD-MT investigators reported a higher proportion of patients achieving 90-day mRS 0–1 with endovascular therapy (EVT) plus best medical management than with best medical management alone in a selected group with anterior-circulation LVO, NIHSS <6, and favorable perfusion imaging. These findings are not directly comparable with TEMPO-2 because the intervention, imaging selection, reperfusion mechanism, time window, and outcome context differed. Short-course dual antiplatelet therapy remains supported for eligible non-cardioembolic minor stroke, while longer-term prevention should be tailored to the identified etiologic subtype. The modified Rankin Scale may miss residual deficits after minor stroke; measures such as the Canadian Outcome Scale for Minor Stroke (COSMOS) may capture performance, participation, and personally meaningful activities more sensitively. The four-domain framework is intended to organize clinical assessment, not to serve as a validated prediction score or treatment algorithm.

## Introduction

Population-based studies have documented a shift toward lower initial stroke severity (1, 2), although stroke still imposes a substantial global burden (3). Current guidance does not support basing acute treatment decisions on the NIHSS score alone (4). Mild or rapidly improving symptoms do not guarantee a favorable course: residual disability has been reported in both untreated and aspirin-treated cohorts (5–7). Early recurrence also remains a concern after minor non-cardioembolic stroke or high-risk transient ischemic attack (TIA) (8, 9). Prospective follow-up has also documented incomplete recovery at 90 days after an initially mild presentation (10).

Definitions differ across trials and registries (11), and the NIHSS itself underweights some cognitive and other functionally important deficits (12, 13). A low score may coexist with large-vessel occlusion (LVO) and collateral-dependent tissue at risk (14–16); the underlying mechanism also affects when deterioration or recurrence occurs (17). Although contemporary studies often use thresholds of 3 or 5 (18, 19), posterior-circulation deficits remain especially liable to underestimation (20, 21). Patients with the same total score may therefore need different imaging, monitoring, and preventive strategies.

Defining a stroke as “minor” by score is only a starting point. Clinicians must also determine whether the persisting deficit is disabling and whether an arterial lesion, threatened tissue, or active source makes the presentation unstable. Accordingly, this review separates mild from nondisabling stroke and organizes risk around three outcomes—END or infarct growth, early recurrent ischemia, and incomplete recovery—before considering the vascular, tissue, and etiologic mechanisms that contribute to them. The four-domain framework is a way to structure this assessment, not a validated risk score.

## Methods

For this narrative review, PubMed was searched from database inception through 30 September 2026. Search terms covered minor, mild, or nondisabling stroke; low NIHSS scores; early neurological deterioration (END) and recurrence; posterior-circulation, lacunar, small subcortical, and branch atheromatous stroke; vascular, collateral, perfusion, and diffusion imaging; thrombolysis, thrombectomy, antiplatelet therapy, and clinical outcomes. Reference lists of key guidelines, systematic reviews, and pivotal trials were also examined. Priority was given to randomized trials, practice guidelines, systematic reviews, large prospective cohorts, registry studies, and mechanistic imaging studies relevant to prognosis or treatment. Because the review was narrative, duplicate screening, a formal risk-of-bias assessment, and meta-analysis were not performed.

## What counts as minor stroke

Minor stroke describes initial clinical severity rather than a single mechanism. NIHSS thresholds improve reproducibility, but they do not identify a uniform subgroup (11, 18, 19). Because the NIHSS is an impairment scale, its total score reflects the pattern and weighting of the items measured (12, 22, 23). In this context, “mild” refers to measured neurological impairment, whereas “nondisabling” is a functional judgment: if the deficit persisted unchanged, it would not be expected to prevent basic activities of daily living or return to work, when applicable (4, 7, 18, 24). These concepts overlap, but global impairment and real-world disability are not interchangeable (25, 26).

Tissue-based TIA definitions classify transient symptoms with imaging-confirmed infarction as ischemic stroke rather than TIA (27). Diffusion-weighted imaging (DWI) frequently identifies acute infarction after a transient or minor event and provides prognostic information beyond the bedside examination (28). In practice, applying this distinction depends on MRI availability and the timing of imaging. A DWI-positive lesion confirms tissue injury, but it does not by itself establish that the deficit is functionally minor, identify the responsible mechanism, or show that no additional tissue remains at risk.

The NIHSS may underestimate posterior-circulation deficits (20, 21). Approximately one fifth of ischemic strokes involve the posterior circulation, yet gait or truncal ataxia, dysphagia, oculomotor abnormalities, and visual field loss may yield a low score (20, 21). A posterior-weighted NIHSS modification improves prognostic discrimination in this setting (29). Dedicated scales can support a syndrome-based examination, although explicit assessment of disability and repeated clinical evaluation remain necessary. TEMPO-2, for example, enrolled patients with NIHSS scores of 0–5 (30). Table 1 compares the operational definitions and assessment approaches that recur in the literature.

**Table 1:** Selected definitions and assessment approaches used in minor stroke research.

Construct	Operational definition/assessment	What it captures	Main limitation
Very mild stroke	NIHSS 0–3, as in CHANCE and POINT (8, 9)	Low measured neurological impairment	May miss disabling focal, posterior, cognitive, or gait deficits
Mild stroke	NIHSS 0–5, common in imaging and reperfusion studies (14, 18, 30)	Broad low-severity trial population	Includes marked clinical, vascular, tissue, and etiologic heterogeneity
Nondisabling stroke	Persisting deficit judged unlikely, if unchanged, to prevent basic activities of daily living or return to work, when applicable (4, 7, 18, 24)	Functional relevance of the persisting deficit	Depends on baseline function, occupation, patient priorities, and clinical judgment
DWI-positive minor/transient event	Transient or minor symptoms with acute infarction on DWI (27, 28)	Confirms ischemic tissue injury	Does not define disability, mechanism, or threatened tissue
Posterior-circulation assessment	NIHSS plus targeted posterior examination; posterior-weighted scale may assist (20, 21, 29)	Detects deficits underweighted by the NIHSS	No adjunct has become a universal replacement for clinical examination
Rapidly improving symptoms	Substantial spontaneous improvement before or during assessment (5, 6)	Describes early clinical trajectory	Residual disability or a high-risk lesion may remain

DWI, diffusion-weighted imaging; NIHSS, National Institutes of Health Stroke Scale. These definitions and approaches address different aspects of minor stroke; none alone establishes a low risk of deterioration or recurrence. “Return to work, when applicable” refers to patients for whom employment was a relevant premorbid role. Examples generally considered disabling include complete hemianopia, severe aphasia, marked neglect, and weakness that prevents sustained antigravity effort.

## Time-dependent risks after minor stroke

The timing of risk after minor stroke varies by outcome (31, 32). END or infarct growth usually appears within hours to days (16, 33–35). Recurrent ischemia is more concentrated over the following days and weeks and is closely tied to the active cause (17, 36). Recovery may remain incomplete for months, while vascular risk persists for years (31, 32, 37). These trajectories should not be compressed into a single judgment of “good” or “poor” prognosis. Treatment-related hemorrhage is a separate outcome, and an unchanged serial examination offers only provisional reassurance. Figure 1 relates the four clinical domains to these time horizons.

**Figure 1:**
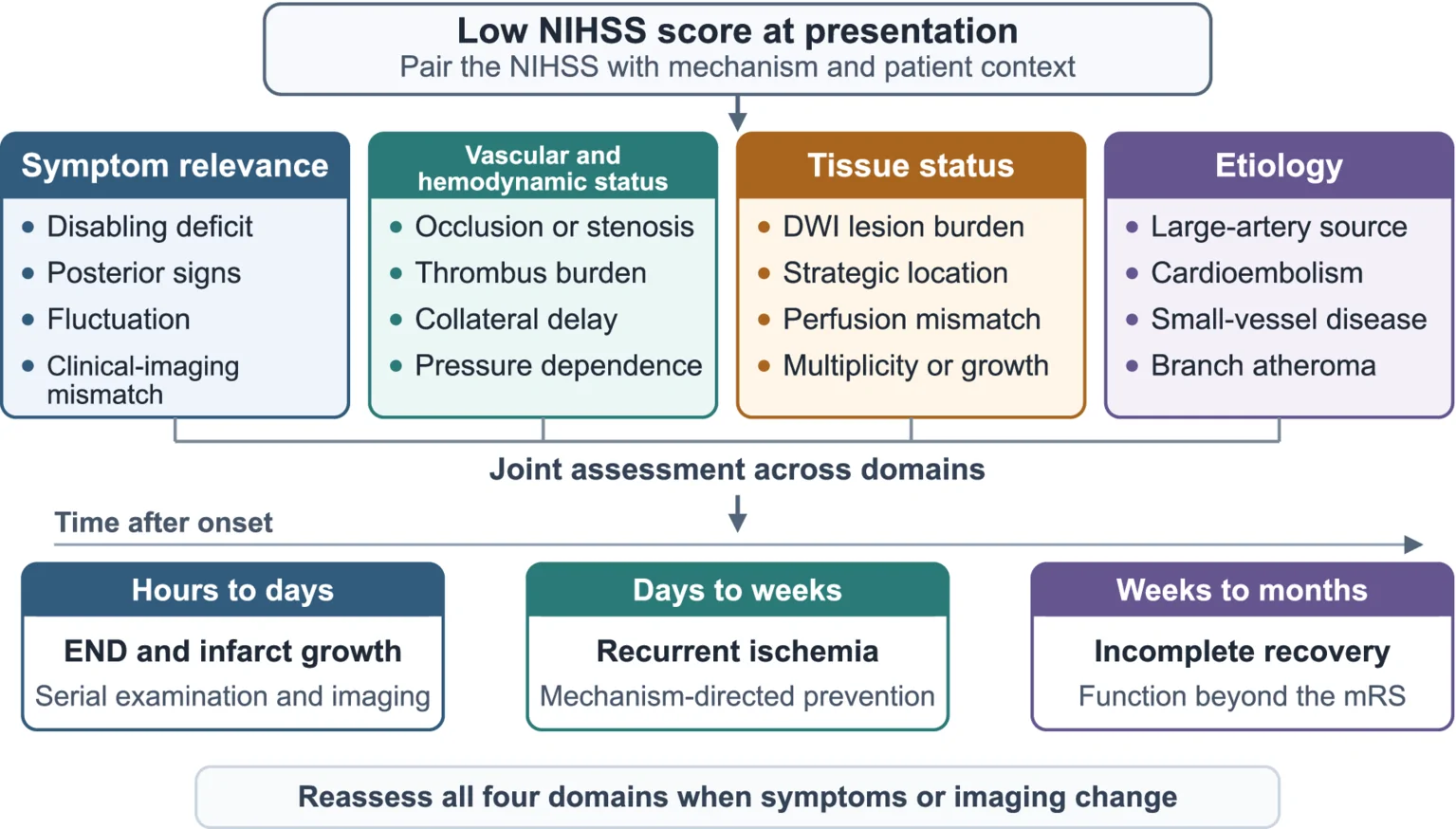
Four-domain, time-dependent framework for minor stroke. Symptom relevance, vascular and hemodynamic status, tissue status, and etiologic subtype are considered together in relation to END or infarct growth, recurrent ischemia, and incomplete recovery. This is a conceptual synthesis based on the reviewed evidence, not a validated score or treatment algorithm; it assigns no weights or treatment thresholds. Treatment-related hemorrhage is considered separately. DWI, diffusion-weighted imaging; END, early neurological deterioration; mRS, modified Rankin Scale; NIHSS, National Institutes of Health Stroke Scale.

An outcome-first approach helps separate prognosis from mechanism. END or infarct growth, early recurrent ischemia, and incomplete recovery are distinct clinical outcomes, whereas LVO, collateral impairment, perfusion deficit, infarct pattern, and etiologic subtype are potential drivers of those outcomes. Keeping these categories separate is particularly important when distinguishing acute vascular instability from recurrence risk related to the underlying cause.

### Symptom relevance

The total NIHSS score does not fully describe the functional importance of a deficit (11–13). Aphasia, dominant-hand weakness, hemianopia, gait instability, dysphagia, or a strategically important cognitive deficit may still be disabling at a low score (19, 24). Baseline independence, work and caregiving demands, and the patient’s priorities provide the necessary context (25, 26). Fluctuation on serial examination may be the first sign that an apparently mild syndrome is unstable. When symptoms appear unexpectedly mild for the arterial occlusion, perfusion abnormality, or lesion location, the clinical and imaging findings should be reassessed. This mismatch alone is not an indication for reperfusion.

### Vascular and hemodynamic status

Vascular assessment should document proximal and distal occlusions, extracranial or intracranial stenosis, tandem disease, thrombus burden, parent-artery plaque, dissection, and collateral status. In minor LVO, the occlusion site is associated with END (14–16). Longer thrombi predict failure of early recanalization, and thrombus extension has been described during otherwise unexplained deterioration (38, 39). The reported prevalence of LVO depends in part on the definition and imaging strategy used (40). Treatment cohorts of mild-deficit LVO have shown variable outcomes (41–43). Distal occlusions and perforator-related lesions can also progress (44–46).

Hemodynamic instability refers to threatened tissue that depends on marginal collateral flow or systemic blood pressure. Clues include symptoms that change with posture or blood pressure, delayed or incomplete collateral filling on multiphase or dynamic CT angiography (CTA), and a perfusion deficit larger than the established infarct. Multiphase CTA samples several time points and can distinguish prompt from delayed collateral filling (47, 48). Stability remains a longitudinal assessment: serial examinations should be paired, when clinically indicated, with repeat evaluation of vessel patency, collateral flow, perfusion, or lesion size. A persistent occlusion can remain clinically stable, but delayed filling or fluctuating symptoms lowers confidence in that judgment.

### Tissue status

Imaging modalities answer different questions. Noncontrast CT excludes hemorrhage and may show a large established infarct, although it is relatively insensitive to small, early, and posterior-fossa lesions. CTA or magnetic resonance angiography defines the arterial lesion, whereas DWI demonstrates acute cytotoxic injury (28). Lesion location may point to a strategic or embolic pattern, and MRI can refine etiologic classification (49). Infarct multiplicity carries additional prognostic information, particularly when intracranial atherosclerotic disease is present (50–52). In a small subcortical infarct, geometry is better used as a mechanistic clue than as a definitive diagnosis.

Fluid-attenuated inversion recovery (FLAIR) hyperintensity generally develops after DWI restriction. DWI–FLAIR mismatch can serve as an imperfect tissue clock for a likely recent infarct, but it does not establish tissue viability (53). CT or MR perfusion estimates contrast transit and blood flow. A region classified as “core” because of low cerebral blood flow is an estimate of tissue likely to infarct, not proof of irreversibility. Mismatch between the estimated core and a larger hypoperfused region is a proxy for potentially salvageable tissue (4). These estimates are most useful when interpreted with the clinical syndrome, vessel imaging, acquisition quality, and the decision under consideration.

A proximal subcortical lesion that reaches the parent artery favors branch atheromatous disease, whereas a small, distal, rounded lesion is more consistent with intrinsic small-vessel disease (45). This distinction can help frame the mechanism of progression and the relevance of parent-artery imaging. It remains a mechanistic inference, however, and should be reconciled with the arterial study and the subsequent clinical course.

### Etiologic subtype

Etiologic assessment asks what produced the index event and whether that cause remains active. Large-artery atherosclerosis, cardioembolism, small-vessel disease, branch atheromatous disease, dissection, and other causes are separated in established etiologic frameworks (36, 54). They differ in the timing and nature of early risk: an untreated source may continue embolization, permit thrombus extension, compromise a perforator origin, or produce a new ischemic event (17, 36). Secondary prevention therefore follows the demonstrated or most likely mechanism rather than the NIHSS score assigned at presentation (55).

Mechanism-focused evaluation begins with arterial imaging and selected laboratory testing. The neutral NAVIGATE ESUS and RE-SPECT ESUS trials do not support empirical anticoagulation for unselected embolic stroke of undetermined source (ESUS) (56–58). Cardiac investigation remains important, and any decision to anticoagulate should rest on a specific indication. Prolonged or implantable rhythm monitoring increases atrial fibrillation detection after ischemic stroke (59–61), and echocardiography can be directed toward patients most likely to have a management-relevant finding (62). The intensity of the investigation should reflect the clinical and imaging phenotype.

### Small-vessel and branch-occlusive mechanisms

A lacunar syndrome does not establish a single arterial mechanism. The lesion may arise from intrinsic small-vessel occlusion, plaque at a perforator origin, or branch atheromatous disease, and imaging patterns can help distinguish these possibilities (44–46). Capsular and pontine warning syndromes comprise recurrent stereotyped lacunar episodes and carry a high short-term risk of completed infarction (63, 64). For this reason, a small subcortical lesion should be described by its topography and relation to the parent artery rather than being treated as mechanistically uniform.

Branch atheromatous disease (BAD) is an important high-risk phenotype even when no proximal LVO is present. Progressive motor deficits and capsular or pontine warning syndromes can reflect instability at the perforator origin and may precede completed infarction (63, 64). Thus, an apparently mild, non-LVO presentation should not be classified as low risk solely because CTA or MRA shows no proximal occlusion. These patients require repeated neurological assessment and mechanism-focused imaging. Evidence for a specific acute rescue strategy remains limited: recent randomized data do not support routine tirofiban for unselected BAD, and observational or subgroup signals require confirmation (65). Management should therefore be individualized according to symptom evolution, infarct pattern, bleeding risk, and the eligibility criteria of available reperfusion or antithrombotic strategies.

## Early neurological deterioration

END denotes worsening attributable to the index stroke, whereas early recurrent ischemia is a new event arising from an active cause. Studies have used different NIHSS thresholds and observation periods, making apparent rates difficult to compare (33–35). An increase of at least 2 NIHSS points is a commonly used definition, but observation windows range from 24 h to several days or longer. Lacunar-stroke reports show similar variation (44). END should therefore be interpreted primarily as a manifestation of acute vascular or tissue instability—such as infarct progression, thrombus propagation, hemodynamic compromise, or perforator-territory extension—whereas stroke etiology is more directly relevant to recurrence prevention. Reporting both the numerical definition and the presumed mechanism of worsening is essential. Etiologic subtype may influence END through these proximal vascular and tissue mechanisms, but it does not itself define END.

END is not confined to LVO. A 2025 systematic review of 67 reports and 13,407 participants found that it occurred in more than one fifth of patients with acute lacunar ischemic stroke, although definitions and observation windows varied (44). Del Bene and colleagues reviewed progressive lacunar stroke, and Mombelli and colleagues observed END in 57 of 292 patients (20%) with small subcortical infarcts (45, 46). Proposed explanations include extension along a perforator territory, branch-origin atheroma, perfusion failure, thrombus propagation, and edema. Because several mechanisms can produce similar clinical worsening, repeat examination should be accompanied by careful lesion characterization.

In minor stroke with a visible arterial occlusion, END has been associated with occlusion site, thrombus length, and failure of early recanalization (16, 38, 39, 41). A low NIHSS score may reflect collateral compensation despite a substantial ischemic burden (14, 15). Multiphase or dynamic CTA can reveal delayed collateral filling that is not apparent from a single arterial phase (47, 48). These patients warrant frequent examination and a predefined rescue pathway (33–35). Observational comparisons have not established that immediate reperfusion benefits every mild presentation (42, 43). Table 2 links the observable findings to the assessment tasks that are most relevant at the bedside.

**Table 2:** Four-domain assessment after minor stroke.

Domain	Observable findings	Primary horizon	Assessment	Clinical purpose
Symptom relevance	Functional impact; eloquent or posterior deficit; fluctuation or warning syndrome; clinical-imaging mismatch	Immediate disability; END over hours to days	Serial examination and explicit functional assessment (11, 19, 24)	Detects disability and instability underweighted by the NIHSS
Vascular and hemodynamic status	Occlusion or stenosis; thrombus burden; parent-artery plaque; collateral delay; pressure dependence	END and infarct growth over hours to days	CTA or MRA for occlusion and END risk (14–16); definition-sensitive LVO classification (40); multiphase CTA or perfusion for collateral timing (47, 48)	Defines the vascular target and hemodynamic reserve
Tissue status	DWI burden and location; multiplicity; perfusion-core mismatch; lesion growth	Infarct growth; incomplete recovery	DWI for acute injury and lesion pattern (28, 49); perfusion when selection-relevant (30); infarct multiplicity (50–52); DWI–FLAIR mismatch (53)	Distinguishes completed injury from threatened tissue
Etiologic subtype	Large-artery, cardiac, intrinsic small-vessel, branch atheromatous, or other cause	Recurrent ischemia over days to weeks; longer-term vascular risk	Arterial and cardiac evaluation (36, 54); small-vessel or branch pattern (44–46); ESUS evidence (56–58); rhythm monitoring (59–61); targeted echocardiography (62)	Directs mechanism-based secondary prevention

CTA, CT angiography; DWI, diffusion-weighted imaging; END, early neurological deterioration; FLAIR, fluid-attenuated inversion recovery; MRA, MR angiography. Findings should be reassessed if the symptoms or imaging phenotype changes. No weights or treatment thresholds have been validated for this table.

## Management strategies

Management decisions differ according to the risk being addressed and its time course. Acute reperfusion seeks to restore flow before irreversible injury. Monitoring and rescue treatment address worsening of the index event, whereas secondary prevention targets recurrent ischemia from the responsible etiologic subtype. Evidence for one of these problems does not necessarily guide treatment of the others. Applying trial results to an individual patient requires explicit attention to eligibility, time window, imaging selection, bleeding risk, the functional importance of the deficit, and the mechanism most likely to drive the relevant outcome.

### Intravenous thrombolysis

Randomized trials in selected minor-stroke populations have not demonstrated superiority or a net benefit of routine intravenous thrombolysis for mild nondisabling deficits. In PRISMS, alteplase did not improve 90-day functional outcome compared with aspirin; symptomatic intracranial hemorrhage occurred in five alteplase-treated patients and no aspirin-treated patients (7). The trial stopped early, clearly disabling deficits were excluded, and vascular imaging was not required for enrollment. Its findings therefore argue against routine alteplase for the population enrolled, but they do not show that every patient with a low NIHSS score lacks a potentially disabling or treatment-responsive deficit. Current guidance retains that distinction (4).

ARAMIS found dual antiplatelet therapy noninferior to alteplase for an excellent 90-day outcome in selected minor nondisabling stroke (18). TEMPO-2 enrolled patients with minor stroke and an intracranial occlusion or a focal perfusion abnormality (30). Tenecteplase did not improve return to baseline function; 90-day mortality was 5% versus 1%, and symptomatic intracranial hemorrhage was numerically more frequent (2% vs. < 1%; RR 4.2, 95% CI 0.9–19.7; *p* = 0.059) (30). A secondary TEMPO-2 analysis found no benefit when an NIHSS-based disabling classification was applied (66). A subsequent TEMPO-2 analysis stratified by baseline vessel status also found no benefit of tenecteplase in complete occlusion, near occlusion, or no visible occlusion with a focal perfusion abnormality (67). Further ARAMIS reports examined END and treatment effects by LVO status and admission NIHSS strata (68, 69), but these were secondary analyses rather than separate randomized trials. The TEMPO-2 findings do not support using occlusion or a focal perfusion abnormality alone as an indication for tenecteplase (30, 66). Table 3 compares the enrolled populations and the principal trial results.

**Table 3:** Randomized trials of acute treatment in minor stroke.

Trial	Population and window	Comparison	Main result	Interpretation
PRISMS (7)	Nondisabling minor stroke; within 3 h	Alteplase vs. aspirin	Did not demonstrate improved 90-day function; symptomatic intracranial hemorrhage increased	Routine alteplase is unsupported in the enrolled population; early termination limits certainty
ARAMIS (18)	Nondisabling minor stroke; within 4.5 h	DAPT vs. alteplase	DAPT was noninferior for an excellent 90-day outcome	DAPT was noninferior in the enrolled nondisabling population; disabling stroke was not addressed
TEMPO-2 (30, 66)	Minor stroke with occlusion or focal perfusion abnormality; within 12 h	Tenecteplase vs. standard care	Did not improve functional outcome; mortality was higher and symptomatic intracranial hemorrhage was numerically more frequent	TEMPO-2 does not support routine tenecteplase in the enrolled imaging-selected population
MILD-MT (74, 75)	Anterior-circulation LVO; NIHSS <6; core ≤50 mL and perfusion mismatch ≥50 mL; within 24 h	EVT plus best medical management vs. best medical management	Positive functional result and less END reported at ESOC 2026; full results article pending	Emerging randomized evidence for a perfusion-selected subgroup; not generalizable to all low-NIHSS LVO

DAPT, dual antiplatelet therapy. Eligibility and outcome definitions differed across trials, so the treatment effects are not directly interchangeable. The MILD-MT efficacy findings shown here are conference-reported; a full peer-reviewed efficacy article remains pending.

### Endovascular treatment in mild-deficit LVO

Most pivotal thrombectomy trials enrolled patients with moderate or severe stroke (70), so their results cannot be transferred directly to mild-deficit LVO. Before 2026, evidence specific to low-NIHSS LVO was predominantly observational and heterogeneous (43, 71–73). MILD-MT was designed as a 300-patient randomized PROBE trial of EVT plus best medical management versus best medical management alone in adults with anterior-circulation LVO, NIHSS <6, ASPECTS ≥6, infarct core ≤50 mL, and perfusion mismatch ≥50 mL within 24 h (74). At ESOC 2026, the investigators reported a positive functional result together with less END in the EVT arm (75). Because the efficacy findings remain conference-reported rather than available as a full peer-reviewed results article, they should be regarded as important emerging randomized evidence rather than as definitive evidence for all patients with mild-deficit LVO.

The contrast between MILD-MT and earlier observational evidence supports investigation of selective, imaging-guided EVT rather than universal intervention for all low-NIHSS LVO. MILD-MT specifically enriched for patients with a substantial volume of salvageable tissue on perfusion imaging (74). By contrast, TEMPO-2 tested intravenous tenecteplase rather than EVT and selected patients by intracranial occlusion or focal perfusion abnormality (30). The different intervention, selection criteria, treatment mechanism, time window, reperfusion process, and outcome context make direct cross-trial comparisons inappropriate. A larger perfusion mismatch and a higher probability of successful reperfusion may plausibly contribute to the positive MILD-MT signal, but the available data do not establish either factor as the sole explanation. A September 2026 TEMPO-2 secondary analysis found no tenecteplase benefit across complete occlusion, near occlusion, or no visible occlusion with focal perfusion abnormality, further arguing against vessel status alone as an explanation for the cross-trial difference (67). Patients not undergoing immediate EVT still require frequent neurological examinations and a predefined rescue pathway when deterioration occurs.

### Early antiplatelet therapy and secondary prevention

For eligible patients with non-cardioembolic minor stroke or high-risk TIA, randomized trials support early dual antiplatelet therapy (DAPT). CHANCE and POINT showed fewer early ischemic events with clopidogrel plus aspirin than with aspirin alone (8, 9). THALES found fewer strokes or deaths with ticagrelor plus aspirin, but severe bleeding was more frequent (76). A systematic review indicates that most benefit from clopidogrel-based DAPT accrues early, whereas bleeding risk continues with longer exposure (77). More recently, a single-center randomized trial with 188 patients in the primary analysis (189 randomized) with supratentorial non-cardioembolic stroke, motor deficits, and no proximal LVO or ≥50% stenosis found less early motor deterioration with loading-dose DAPT than with non-loading antiplatelet therapy; however, serious bleeding events occurred and multicenter confirmation is needed (78). For eligible patients, guidelines favor a short early course, with the agent and duration determined by mechanism, timing, and bleeding risk (4, 55). Table 4 summarizes the pivotal randomized trials informing early recurrence prevention.

**Table 4:** Randomized trials informing early recurrence prevention.

Trial	Population and window	Comparison	Efficacy	Safety and use
CHANCE (9)	Minor non-cardioembolic stroke or high-risk TIA; within 24 h	Clopidogrel plus aspirin vs. aspirin	Reduced 90-day stroke recurrence	Major hemorrhage was not increased in the trial population; supports a short early course
POINT (8)	Minor ischemic stroke or high-risk TIA; within 12 h	Clopidogrel plus aspirin vs. aspirin	Reduced major ischemic events	Major hemorrhage increased; most efficacy accrued early while bleeding risk continued
THALES (76)	Nonsevere ischemic stroke or high-risk TIA; within 24 h	Ticagrelor plus aspirin vs. aspirin	Reduced stroke or death at 30 days	Severe bleeding increased; use follows trial eligibility and local guidance

TIA, transient ischemic attack. Eligibility, regimen, treatment window, and bleeding definitions differed across these prevention trials. This table is limited to early dual-antiplatelet trials in non-cardioembolic minor stroke or high-risk TIA; BAD/tirofiban trials are discussed separately in the text.

### Etiology-specific treatment considerations

The treatment implications of minor stroke differ by etiologic subtype and should not be reduced to a single antithrombotic strategy. For large-artery atherosclerosis, early antiplatelet therapy is appropriate for eligible non-cardioembolic events, while the arterial lesion itself should guide evaluation for extracranial or intracranial stenosis and other mechanism-specific prevention. For cardioembolic stroke, identification of a definite cardiac source changes the preventive strategy toward anticoagulation when indicated; antiplatelet therapy should not be used as an empirical substitute for an established cardioembolic indication. For intrinsic small-vessel disease, antiplatelet therapy and vascular risk-factor management remain central, while the presence of BAD or a warning syndrome warrants closer observation because early progression can occur without proximal LVO. In BAD, randomized evidence does not currently support routine tirofiban, so treatment should remain individualized and linked to the studied imaging phenotype and bleeding risk (55, 63, 65, 79).

### Branch atheromatous disease and tirofiban

RESCUE BT2 was conducted in China and found a higher proportion of excellent 90-day functional outcomes with intravenous tirofiban than with aspirin (79). The trial did not include patients with large- or medium-vessel occlusion. Entry required an NIHSS score of at least 5 and at least one limb with moderate-to-severe weakness (NIHSS motor item score 2–4), together with one of four prespecified clinical presentations. Most infarcts were presumed atherosclerotic. These eligibility criteria define a selected non-LVO population rather than unselected minor stroke. Its result should not be used to infer benefit for every low-NIHSS presentation or for branch atheromatous disease defined by other imaging criteria.

STRATEGY more directly addressed branch atheromatous disease. The trial enrolled 970 patients in China with MRI-confirmed BAD and compared tirofiban plus aspirin with aspirin alone (65). The primary composite of END within 7 days or new stroke within 90 days was not significantly reduced (17.1% vs. 19.6%; HR 0.88, 95% CI 0.65–1.19), and moderate or severe bleeding was uncommon. Disability or death at 90 days (mRS 2–6) was lower in the tirofiban group as a secondary outcome, but this finding should be interpreted in the context of the neutral primary endpoint. Current randomized evidence therefore does not support routine tirofiban for unselected BAD; whether narrower imaging-defined subgroups benefit remains uncertain.

### Blood pressure and hemodynamic management

Outside established reperfusion thresholds, no minor stroke-specific trial has defined an optimal blood-pressure target, so established acute-stroke guidance remains the reference (4). Precipitous reductions should be avoided when symptom fluctuation, collateral delay, or a perforator-territory syndrome suggests pressure-dependent perfusion (4). Multiphase vascular imaging can help identify delayed collateral filling (47, 48). Hypotension and other reversible causes of hypoperfusion require correction, followed by neurological reassessment after a clinically important pressure change (4). Avoiding an abrupt reduction does not justify induced hypertension or leaving severe hypertension untreated. Lacunar and small-vessel guidance likewise supports mechanism-aware management rather than an untested minor-stroke target (63). Figure 2 places blood-pressure assessment within the broader sequence of acute decisions.

**Figure 2:**
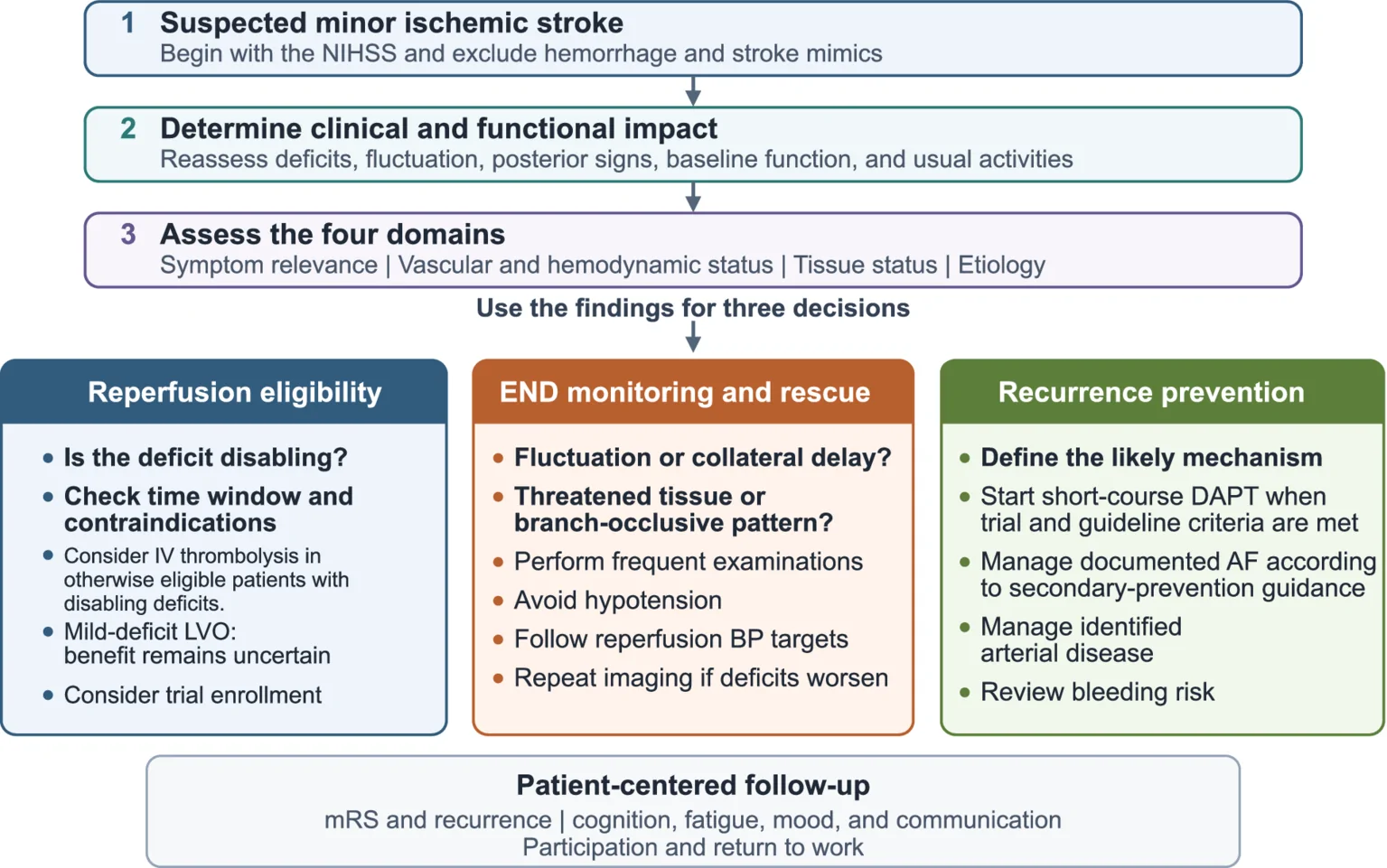
Integrated decision pathway for suspected minor ischemic stroke. A low NIHSS score prompts explicit assessment of symptom relevance, vascular and hemodynamic status, tissue status, and etiology before decisions about reperfusion, monitoring for END, and prevention of recurrent ischemia are made. This pathway summarizes the reviewed evidence and is intended to support, rather than replace, established eligibility criteria, contraindication assessment, and local acute-stroke protocols. AF, atrial fibrillation; BP, blood pressure; DAPT, dual antiplatelet therapy; END, early neurological deterioration; IV, intravenous; LVO, large-vessel occlusion; mRS, modified Rankin Scale; NIHSS, National Institutes of Health Stroke Scale.

## Discussion

Similar NIHSS scores can occur in very different clinical settings: a stable small infarct, collateral-dependent LVO, evolving perforator ischemia, or an active embolic process. The immediate concern also differs. The index lesion may enlarge, a new ischemic event may occur, or a seemingly modest deficit may prevent return to premorbid activities. Clinical findings, vessel status, tissue imaging, and etiology are therefore most useful when interpreted against a defined outcome and time horizon. The aim is not to label every patient as simply “high risk,” but to identify the outcome at risk and the mechanism most likely to drive it.

### Outcome assessment

The modified Rankin Scale (mRS) remains important, but dichotomized scores can miss meaningful deficits after minor stroke (25, 26). A patient may remain ambulatory and independent yet fail to resume premorbid activities or work. Fatigue is common after TIA or minor stroke (80, 81), and longer-term cohorts identify persistent cognitive and physical limitations (82, 83). Depression is also associated with disability and quality of life (84). These outcomes become particularly important when baseline impairment and the expected treatment effect are small, because a conventional “good outcome” can still conceal a change that matters to the patient. The need for more sensitive outcome measures has led to development of minor-stroke-specific tools such as COSMOS (85).

### Evidence limitations and unresolved controversies

Acute-treatment trials have not used a uniform definition of minor or nondisabling stroke (7, 18, 30). The distinction should be based on the functional consequences of a persisting deficit rather than NIHSS score alone; contemporary guidance emphasizes whether the patient can perform basic activities of daily living and, where relevant, return to work, with specific deficits such as complete hemianopia, severe aphasia, marked neglect, or weakness that prevents sustained antigravity effort generally considered disabling (4). For END, both the threshold and the observation window differ between studies (33, 34, 44). Reperfusion trials enrolled selected populations rather than every patient with a low NIHSS score (7, 18, 30, 70). MILD-MT now provides emerging randomized evidence for EVT in a narrowly selected, perfusion-enriched mild-LVO population (74, 75), but the full peer-reviewed efficacy report remains pending and the findings should not be generalized to all mild-deficit LVO.

Imaging criteria for branch atheromatous disease are not uniform (44–46, 63). The relevant tirofiban trials enrolled selected Chinese populations (65, 79). Differences in mechanism, background therapy, and practice limit direct generalization (50, 55, 63). The literature search for this narrative review was targeted rather than systematic, and the four-domain approach has neither prospective validation nor predictive weights. The framework should therefore be used to organize a case or study design, not as a prospectively validated predictive tool.

### Research priorities

Trials based only on an NIHSS threshold risk mixing patients with different arterial lesions and mechanisms. Mild-deficit LVO studies should prespecify occlusion site, thrombus burden, collateral status, perfusion pattern, functional relevance, and the rescue strategy available to the control group. Studies of small subcortical stroke should distinguish intrinsic small-vessel disease from parent-artery plaque or branch atheromatous disease (44–46) and use a prespecified definition of END (63). Future trials should also specify the clinically meaningful outcome being targeted—END, recurrent ischemia, or incomplete recovery—rather than treating “poor outcome” as a single construct. Prespecifying these variables would make study results easier to interpret without assuming a common treatment response across all low-score presentations.

Risk models should estimate END, recurrent ischemia, treatment-related hemorrhage, and incomplete recovery separately, because their predictors and timing differ. Imaging and mechanism variables need prospective validation rather than *post hoc* weighting. Blood biomarkers are being studied for diagnosis and early deterioration (86–88), and measures of brain frailty may add prognostic information within apparently minor stroke (89). Their incremental value must be tested against readily available clinical and imaging data before they are used to guide treatment. At present, neither class of marker is a substitute for arterial imaging, serial examination, or mechanism-focused investigation.

Minor-stroke-specific outcome measures may capture this residual burden more fully. The Canadian Outcome Scale for Minor Stroke (COSMOS) is a 7-point scale ranging from no symptoms to loss of independence and includes limitations affecting hobbies, employment, education, service, and caregiving. Its initial reliability study showed substantial interrater and almost-perfect intrarater agreement, although prospective validation remains necessary (85). COSMOS may therefore complement the mRS by capturing domains that matter after minor stroke, but it has not yet replaced established trial endpoints. The study cited here assessed reliability; COSMOS has not yet been validated as a primary endpoint in an interventional trial.

Outcome measurement should reflect when each event occurs. END, infarct growth, and treatment-related harm belong to the acute phase, while recurrent vascular events and recovery in cognition, fatigue, mood, communication, participation, and work require later assessment. A single 90-day disability endpoint cannot describe this full course. Long-term follow-up is also warranted because cumulative stroke risk remains clinically important for 5 to 10 years after TIA or minor stroke (17, 37). Trial endpoints and follow-up intervals should match the event being studied.

## Conclusion

Minor ischemic stroke should not be treated as a uniformly low-risk condition. NIHSS remains useful for describing initial neurological severity, but treatment and monitoring also depend on the functional importance of the deficit, arterial pathology, tissue vulnerability, and etiologic subtype. An outcome-first approach—separating END or infarct growth, recurrent ischemia, and incomplete recovery—helps link each risk to the mechanism most likely to produce it. Short-course DAPT is supported for eligible non-cardioembolic events, whereas other preventive and acute strategies should be tailored to the identified mechanism and to established trial or guideline eligibility. Routine thrombolysis has not shown net benefit in the selected populations with mild nondisabling deficits studied to date. MILD-MT offers emerging randomized evidence for EVT in a perfusion-selected subgroup with mild-deficit anterior-circulation LVO, but the efficacy results remain conference-reported and should not be generalized to all low-NIHSS LVO. Outcome assessment should also extend beyond conventional mRS categories to cognition, fatigue, participation, work, and other patient-relevant domains. COSMOS is one candidate measure, but further validation is needed.
